# Necrobiosis lipoidica: a rare complication of diabetes

**DOI:** 10.11604/pamj.2018.29.214.15550

**Published:** 2018-04-13

**Authors:** Nassiba Elouarradi, Nawal El Ansari

**Affiliations:** 1 Service of Endocrinology, Diabetology and Metabolic Diseases, University Hospital of Marrakech, Marrakech, Morocco

**Keywords:** Necrobiosis, lipoidica, diabetes

## Image in medicine

We report the case of a 21-year-old patient, known as a type 1 diabetic for 11 years on insulin therapy, who for the past 4 years has had a skin lesion that is progressively increasing in size on the anterior aspect of the left leg. The clinical examination revealed a sclero-atrophic cupboard with erythematous border on the anterior aspect of the leg. A skin biopsy was made revealing a morphological appearance compatible with necrobiosis lipoidica. Necrobiosis Lipoidica is a rare cutaneous manifestation, the prevalence of which is estimated at 0.3% in diabetics. The lesions are characterized by confluent papules in irregular patches, mainly on the anterior surface of the legs, installed bilaterally and symmetrically. Other locations are rarer: scalp, face, arm and trunk. The edges are infiltrated. The lesions evolve most often towards a central ulceration. The pathophysiology is hypothetical and the lesions could be due to cutaneous microangiopathy. It is associated with type 1 or 2 diabetes, excluding an autoimmune origin. Therapeutically, remission can be spontaneous. No treatment has been shown to be effective. Local corticosteroids under occlusion are proposed for recent lesions, but not for atrophic lesions as they may precipitate ulceration.

**Figure 1:**
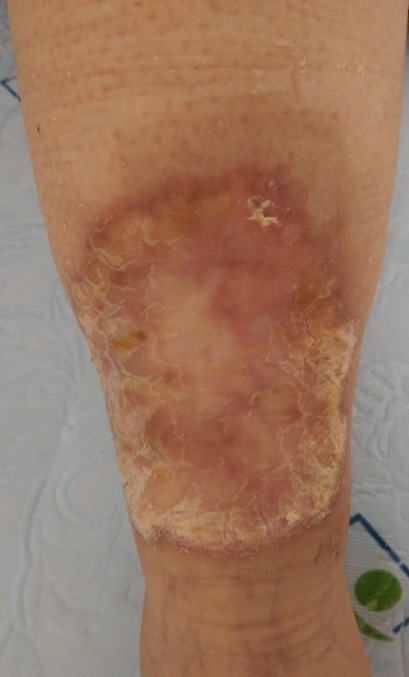
Necrobiosis lipoidica on the anterior aspect of the left leg

